# Low Cholinesterase Is a Potential Poor Prognostic Factor in Colorectal Cancer Presenting With Tumor Markers Negative

**DOI:** 10.1002/cnr2.70266

**Published:** 2025-08-01

**Authors:** Tawfik Ali Hamood Alburiahi, Lei Liang, Weiqing Liu, Zhiyong Kou, Yunfei Zhang, Ning Xu, Jun Yang

**Affiliations:** ^1^ Department of Surgical Oncology The First Affiliated Hospital of Kunming Medical University Kunming China; ^2^ Kunming Medical University Kunming China; ^3^ Department of Internal Medicine‐Oncology The First Affiliated Hospital of Kunming Medical University Kunming China

**Keywords:** butyrylcholinesterase, cholinesterase, colorectal cancer, prognosis, tumor markers negative

## Abstract

**Background:**

Colorectal cancer (CRC) is the third most common malignant tumor in the world and has the second highest mortality rate. Tumor markers are proteins used to diagnose and monitor cancer. Serum cholinesterase (CHE) is a nutritional indicator indicating the liver''s ability to synthesize proteins and is a predictor of CRC. Butyrylcholinesterase (BCHE), a CHE enzyme encoded by the BCHE gene, is synthesized by the liver and released into the serum.

**Objective:**

To study the association between CHE and survival prognosis in CRC with tumor markers negative (TMN). The relationship between the BCHE gene and immune cell infiltration was also explored.

**Methods:**

The clinical data of patients with CRC were collected. The data included tumor markers and biochemical indicators. Patients were divided into different groups for prognosis analysis. CHE levels were used as the cutoff for classification. Further analysis was conducted on the all‐TMN group. CHE was found to be correlated with survival prognosis. This study also analyzed BCHE gene expression in cancer and normal tissues. A correlation analysis was conducted with other factors.

**Results:**

(1) Among 1140 patients who met the criteria, the TMN group (*n* = 369) had a higher survival rate (89.7%) than the TMP group (*n* = 771, 83.3%; *p* = 0.035). (2) Among 1140 CHE, the low cholinesterase (CHE^Low^) group (*n* = 165) had worse survival (73.9%) compared to the high cholinesterase (CHE^High^) group (*n* = 975, 87.3%; *p* < 0.001). (3) In the TMN group, CHE^Low^ (*n* = 48) had worse survival (79.2%) than CHE^High^ (*n* = 321, 91.3%; *p* = 0.008). Similarly, in the TMP group, CHE^Low^ (*n* = 117) showed poorer survival (71.8%) compared to CHE^High^ (*n* = 654, 85.3%; *p* < 0.001). (4) In colorectal cancer with all TMN, CHE ≤ 5.4 U/L, BMI < 18.5 kg/m^2^, and pN2 were independent detrimental prognostic factors for overall survival (OS) (*p* < 0.05). (5) BCHE expression differs between cancer and normal CRC tissues. BCHE expression correlated with pathological stage and progression‐free survival. BCHE expression positively correlated with immune cell infiltration (*p* = 2.7e^−28^, *r* = 0.59), distinctively M2 macrophage infiltration (*p* < 0.0001).

**Conclusion:**

In CRC with TMN, CHE is an independent detrimental prognostic factor. BCHE may serve as a biomarker for CRC to help predict its prognosis and may have an essential impact on immunotherapy.

**Abbreviations**
AFP: Alpha‐fetoprotein
BCHE: Butyrylcholinesterase
BMI: Body mass index
CA125: Carbohydrate antigen 125
CA153: Carbohydrate antigen 153
CA19‐9: Carbohydrate antigen 19‐9
CEA: Carcinoembryonic antigen
CHE: Serum cholinesterase
CHE^High^
: High cholinesterase
CHE^Low^
: Low cholinesterase
CI: Confidence interval
CRC: Colorectal cancer
GTEx: Genotype‐tissue expression
TCGA: The cancer genome atlas
TIMER: Tumor immune estimation resource
TMN: Tumor marker negative
TMP: Tumor marker positive

## 1 Introduction

Colorectal cancer (CRC) is the third most common malignant tumor in the world and has the second highest mortality rate [1]. Tumor markers are proteins and enzymes detected in blood or other bodily fluids and are often associated with cancer [2]. These markers are frequently used to aid in cancer diagnosis, prognosis, and monitoring. In CRC, common markers include alpha‐fetoprotein (AFP), carcinoembryonic antigen (CEA), carbohydrate antigens (CA125, CA153, and CA19‐9) [3, 4]. Tumor markers positive (TMP), elevated levels of these markers may indicate CRC or disease progression. However, some patients are diagnosed with CRC despite tumor markers negative (TMN), highlighting the need to identify additional risk factors for improved screening and prognosis.

Cholinesterase (CHE) is primarily synthesized in hepatocytes and released into the bloodstream [5]. Its activity declines in cases of hepatic dysfunction due to impaired synthetic capacity. Serum CHE levels have emerged as significant prognostic indicators not only in hepatocellular carcinoma (HCC) but also in various other malignancies [6]. Studies have demonstrated its predictive value in conditions such as sarcopenia [7], gastric [8], and pancreatic [9] cancers. Butyrylcholinesterase (BCHE) is the CHE enzyme encoded by the BCHE gene. It belongs to the type‐B carboxylesterase/lipase protein family (provided by RefSeq, Jul 2016), present in the nervous system and liver [10]. Serum BCHE levels are known to decrease in several clinical conditions, including liver damage and malignancies [11]. Research has also shown correlations between BCHE and key biological processes such as tumorigenesis [12], breast cancer [13], and colorectal neoplasia [14]. CHE may serve as potential protein markers in certain tumors.

To our knowledge, no studies have examined the prognostic value of CHE in CRC with TMN. This study aims to investigate the association between CHE and survival outcomes in CRC with TMN and explore the relationship between BCHE gene expression and immune cell infiltration in CRC.

## 2 Materials and Methods

### 2.1 Patients

A retrospective study was conducted by selecting and collecting clinicopathological data from 1140 CRC patients diagnosed with adenocarcinoma at the First Affiliated Hospital of Kunming Medical University between January 2014 and September 2019. The patient cohort was established following specific inclusion and exclusion criteria, as illustrated in the flow chart (Figure S1).

### 2.2 Differential Expression (DE) Analyses

The standard reference values for AFP, CEA, CA125, CA153, and CA19‐9 were used as thresholds. Those who were all negative for the above tumor markers were included in the TMN group, and those who were positive for any of the above tumor markers were included in the TMP group. The optimal cut‐off value for CHE was determined using X‐tile software (version 3.6.1; Figure S3). Patients were divided into low (≤ 5.4 U/L) and high (> 5.4 U/L) cholinesterase (CHE^Low^; CHE^High^) groups based on a 5.4 U/L cutoff. The process for identifying the study participants is detailed in the flowchart (Figure S2).

### 2.3 Survival Analysis

The chi‐square test was employed to compare the distribution of individual variables between patient groups. Receiver operating characteristic (ROC) curve analysis was performed to calculate the area under the curve (AUC) for survival analysis. Survival curves were generated using the Kaplan–Meier method, and differences between curves were assessed with the log‐rank test. Univariate and multivariate Cox regression analyses were conducted to evaluate prognostic factors for overall survival (OS) using Cox's proportional hazards ratio (HR), a 95% confidence interval (CI), and a stepwise approach. A *p*‐value of < 0.05 was considered statistically significant. All statistical analyses were performed using SPSS Statistics version 27.0 (SPSS Inc., Chicago, IL, USA). Survival figures and graphs were drawn using the GraphPad Prism software (version 8.0.2).

### 2.4 Functional Analysis

We conducted a differential expression analysis to compare high and low CHE in CRC patients and performed a survival prognosis analysis. Kaplan–Meier survival analysis was used to evaluate the relationship between CHE and the survival time of CRC patients, with statistical significance defined as a *p*‐value < 0.05.

Additionally, conventional clinicopathological factors (e.g., sex, age, body mass index (BMI), mismatch repair (MMR) status, primary tumor site, tumor type, tumor differentiation degree, tumor size, pT stage, pN stage, M stage, vascular invasion, nerve invasion, and preoperative intestinal obstruction) and biochemical indicators (e.g., neutrophil‐to‐lymphocyte ratio (NLR), lymphocyte‐to‐monocyte ratio (LMR), platelet‐to‐lymphocyte ratio (PLR), and CHE) were analyzed in the TMN group. Cox regression survival analysis was performed for all patients in all tumor markers and the TMN group. Univariate analysis, including HR, 95% CI, and prognosis evaluation, was followed by multivariate analysis using a conditional forward stepwise regression method to identify independent prognostic factors. Propensity score matching (1:1) was performed to balance baseline characteristics between CHE low and high groups (Table S7).

### 2.5 BCHE Related Analysis

We obtained standardized pan‐cancer data from the UCSC Xena database (https://xenabrowser.net/), The Cancer Genome Atlas (TCGA), Genotype‐Tissue Expression (GTEx), and extracted BCHE gene expression for each sample [15]. Using R (v3.6.4), we analyzed differences in BCHE expression between tumor and normal tissues and across clinical stages. Statistical tests included unpaired student's *t*‐test and Wilcoxon rank‐sum test. The R package maxstat was used to find the optimal BCHE expression cutoff (−1.5951), splitting patients into high and low expression groups, Sangerbox (http://www.sangerbox.com/tool). Survival analysis was done using the survival package, with significance tested via the log‐rank test. We also analyzed tumor gene expression data using GeneSymbol and calculated stromal, immune, and ESTIMATE scores with the ESTIMATE package (version 1.0.1) [16]. Pearson correlations between gene expression and immune infiltration scores were assessed using psych (version 2.1.6) [16]. The expression level of 0 and performed a log2 (*x* + 0.001) transformation on each expression value was filtered. According to gene expression, the R software package IOBR (version 0.99.9) [17] deconvo_quantiseq method (QUANTISEQ) [18] and the timer method (TIMER) [19] are used to perform immune deconvolution.

## 3 Result

### 3.1 Patient Characteristics

**FIGURE 1:**
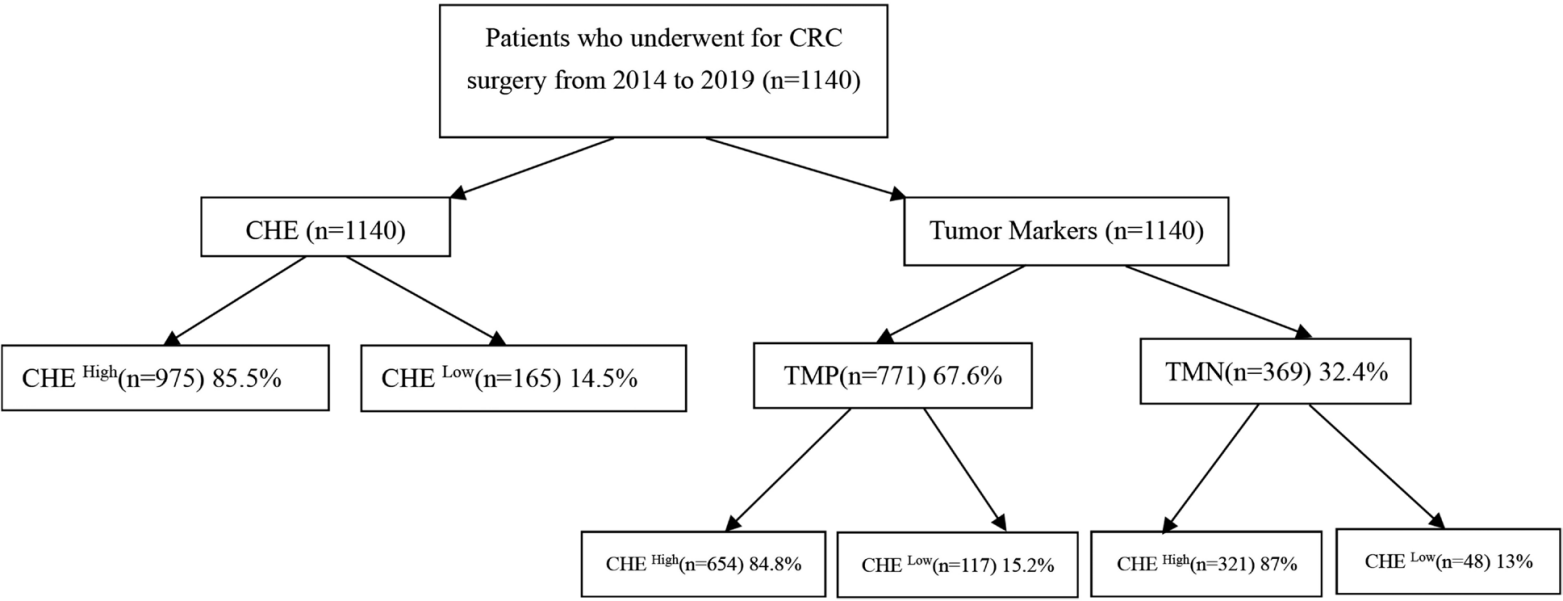
Flowchart of grouping patients and definition of study.

Among the 1140 CRC patients included in the study, 369 (32.4%) in the TMN group and 771 (67.6%) in the TMP group (Figure 1). The TMN group comprised 153 (33.2%) female and 216 (31.8%) male patients, whereas the TMP group included 308 (66.8%) females and 463 (68.2%) males. Baseline characteristics of the patients, categorized by tumor markers in CRC, are detailed in (Table 1).

**TABLE 1:** Patients' characteristics according to all tumor markers in CRC.

Variables	Subset		All tumor markers^a^ (*n* = 1140)		
			Negative markers (*n* = 369) (32.4%)	Positive markers (*n* = 771) (67.6%)	*p*
Sex	Female	(*n* = 461)	153 (33.2%)	308 (66.8%)	0.626
	Male	(*n* = 679)	216 (31.8%)	463 (68.2%)	
Age (yr)	(< 70)	(*n* = 839)	292 (34.8%)	547 (65.2%)	0.003
	(≥ 70)	(*n* = 301)	77 (25.6%)	224 (74.4%)	
BMI (kg/m^2^)	(< 18.5)	(*n* = 92)	28 (30.1%)	65 (69.9%)	0.627
	(≥ 18.5)	(*n* = 1046)	341 (32.6%)	706 (67.4%)	
MMR	0	(*n* = 935)	299 (32.0%)	636 (68.0%)	0.548
	1	(*n* = 205)	70 (34.1%)	135 (65.9%)	
NLR	(< 3.3)	(*n* = 914)	301 (32.9%)	613 (67.1%)	0.413
	(≥ 3.3)	(*n* = 226)	68 (30.1%)	158 (69.9%)	
LMR	(< 3)	(*n* = 340)	99 (29.1%)	241 (70.9%)	0.126
	(≥ 3)	(*n* = 800)	270 (33.8%)	530 (66.3%)	
PLR	(< 230.5)	(*n* = 909)	299 (32.9%)	610 (67.1%)	0.452
	(≥ 230.5)	(*n* = 231)	70 (30.3%)	161 (69.7%)	
CHE	(≤ 5.4 U/L)	(*n* = 165)	48 (29.1%)	117 (70.9%)	0.331
	(> 5.4 U/L)	(*n* = 975)	321 (32.9%)	654 (67.1%)	
pT‐stage	1	(*n* = 51)	25 (49.0%)	26 (51.0%)	< 0.001
	2	(*n* = 223)	87 (39.0%)	136 (61.0%)	
	3	(*n* = 599)	195 (32.6%)	404 (67.4%)	
	4	(*n* = 267)	62 (23.2%)	205 (76.8%)	
pN‐stage	0	(*n* = 656)	234 (35.7%)	422 (64.3%)	0.020
	1	(*n* = 325)	92 (28.3%)	233 (71.7%)	
	2	(*n* = 159)	43 (27.0%)	116 (73.0%)	
pM‐stage	0	(*n* = 1070)	358 (33.5%)	712 (66.5%)	0.002
	1	(*n* = 70)	11 (15.7%)	59 (84.3%)	
Vascular invasion	0	(*n* = 885)	306 (34.6%)	579 (65.4%)	0.003
	1	(*n* = 255)	63 (24.7%)	192 (75.3%)	
Nerve invasion	0	(*n* = 662)	239 (36.1%)	423 (63.9%)	0.002
	1	(*n* = 478)	130 (27.2%)	348 (72.8%)	
Obstructive	0	(*n* = 1043)	345 (33.1%)	698 (66.9%)	0.093
	1	(*n* = 97)	24 (24.7%)	73 (75.3%)	
Tumor size	(< 3.8 cm)	(*n* = 544)	198 (36.4%)	346 (63.6%)	0.005
	(≥ 3.8 cm)	(*n* = 596)	171 (28.7%)	425 (71.3%)	
Location of cancer	Colon	(*n* = 581)	177 (30.5%)	404 (69.5%)	0.161
	Rectum	(*n* = 559)	192 (34.3%)	367 (65.7%)	
Differentiation	Well	(*n* = 530)	188 (35.5%)	342 (64.5%)	0.089
	Moderately	(*n* = 467)	142 (30.4%)	325 (69.6%)	
	Poorly	(*n* = 143)	39 (27.3%)	104 (72.7%)	

^a^ All tumor markers (AFP, CEA, CA125, CA153, and CA19‐9) were used as boundaries. The group that did not have any of the aforementioned tumor markers was known as the tumor marker negative group. The group that had any of the tumor markers was known as the positive group.

### 3.2 Survival Outcome

#### 3.2.1 Tumor Markers (Negative vs. Positive) are Associated With Survival in CRC

**FIGURE 2:**
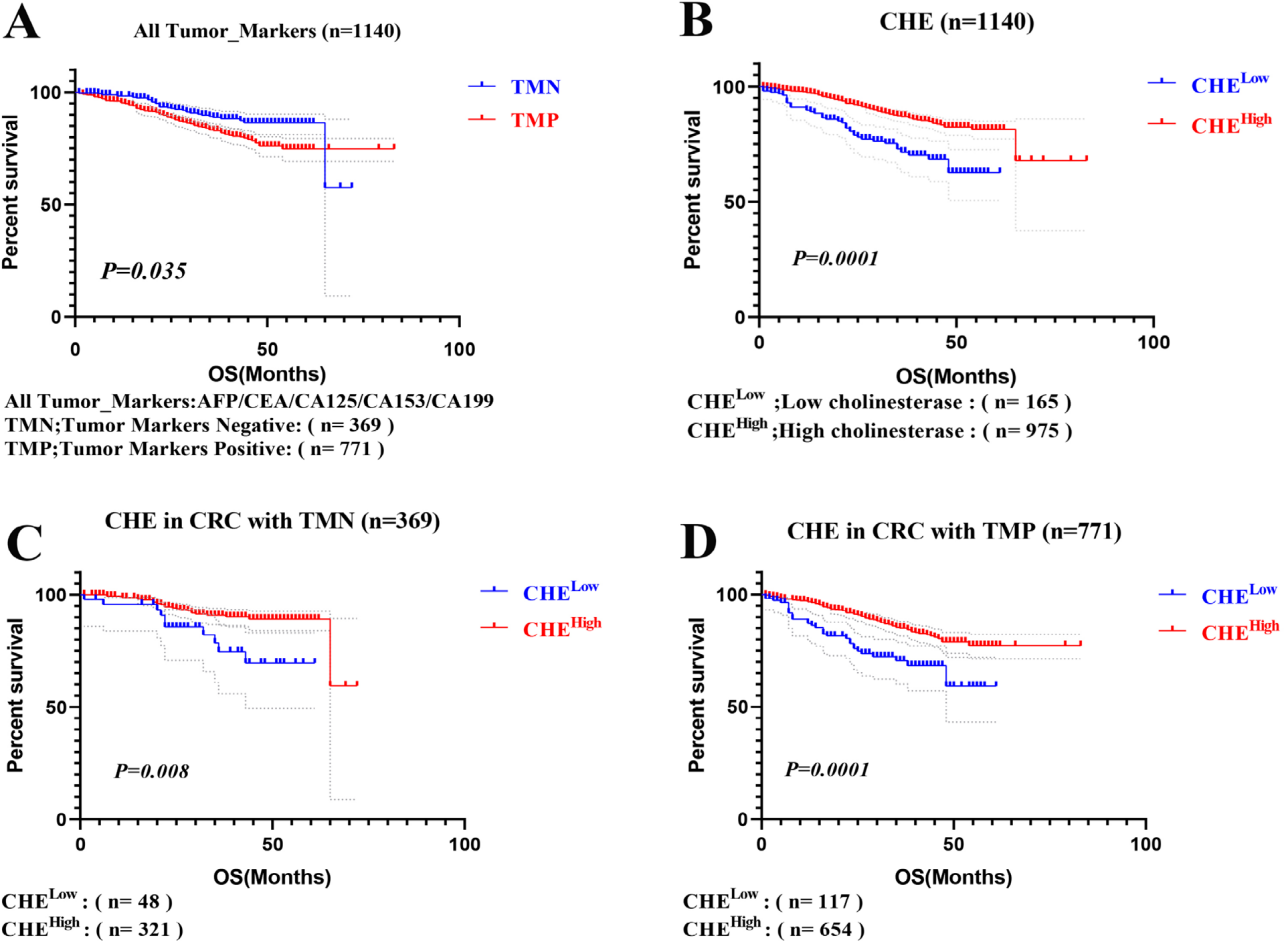
Kaplan–Meier survival analysis of tumor markers and CHE in CRC patients. (A) Patients with TMP show significantly worse OS than those with TMN (*p* = 0.035). (B) CHE^Low^ are associated with significantly reduced OS compared to CHE high (*p* = 0.0001). (C) Among patients with TMN, those with CHE^Low^ have poorer OS than those with CHE^High^ (*p* = 0.008). (D) Among patients with TMP, low CHE levels are also linked to worse OS (*p* = 0.001). Therefore, CHE^Low^ is independently associated with poor prognosis in CRC patients, highlighting their potential value as prognostic indicators; low cholinesterase (CHE^Low^); high cholinesterase (CHE^High^); tumor markers positive (TMP); tumor markers negative (TMN); overall survival (OS); CHE ^Low^ ≤ 5.4 U/L; CHE ^High^ > 5.4 U/L. The dashed lines represent the confidence intervals (95% CI).

Univariate and multivariate Cox regression analyses were conducted to evaluate the prognostic significance of CHE in conjunction with other clinicopathological parameters. In the univariate analysis, significant prognostic factors included sex (male vs. female; HR 1.43, 95% CI: 1.038–1.969, *p* < 0.029), age (years; HR 0.484, 95% CI: 0.353–0.663, *p* < 0.001), BMI (HR 2.204, 95% CI: 1.44–3.373, *p* < 0.001), LMR (HR 1.668, 95% CI: 1.22–2.281, *p* = 0.001), CHE (HR 2.267, 95% CI: 1.602–3.209, *p* < 0.001), and tumor markers (negative vs. positive) (HR 0.559, 95% CI: 0.389–0.803, *p* < 0.002). The association between tumor marker status and OS in CRC patients is summarized in (Table 2). In the multivariate analysis, significant prognostic factors included sex (HR 1.424, 95% CI: 1.03–1.968, *p* = 0.032), age (HR 0.496, 95% CI: 0.36–0.683, *p* < 0.001), BMI (HR 2.675, 95% CI: 1.726–4.146, *p* < 0.001), LMR (HR 1.555, 95% CI: 1.13–2.14, *p* = 0.007), and tumor marker (HR 0.675, 95% CI: 0.468–0.972, *p* = 0.035). These remained significant prognostic factors (Table 2). Kaplan–Meier analysis further revealed a significant difference in survival rates, with patients having TMN showing a higher survival rate (89.7%) compared to those with TMP (83.3%). This difference was statistically significant (*p* < 0.035) and is illustrated in (Figure 2A).

**TABLE 2:** Tumor marker status (negative vs. positive) and survival in CRC: univariate/multivariate results.

Variables	Univariate analysis		Multivariate analysis	
	HR (95% CI)	*p*	HR (95% CI)	*p*
Gender				
Male	1.43 (1.038–1.969)	0.029	1.424 (1.03–1.968)	**0.032**
Female	Reference			
Age				
< 70	0.484 (0.353–0.663)	0	0.496 (0.36–0.683)	**0**
≥ 70	Reference			
BMI				
< 18.5	2.204 (1.44–3.373)	0	2.675 (1.726–4.146)	**0**
≥ 18.5	Reference			
MMR				
pMMR	1.309 (0.848–2.018)	0.224		
dMMR	Reference			
NLR				
< 3.3	0.607 (0.431–0.854)	0.004		
≥ 3.3	Reference			
LMR				
< 3	1.668 (1.22–2.281)	0.001	1.555 (1.13–2.14)	**0.007**
≥ 3	Reference			
PLR				
< 230.5	0.673 (0.476–0.953)	0.026		
≥ 230.5	Reference			
CHE				
≤ 5.4	2.267 (1.602–3.209)	0		
> 5.4	Reference			
Tumor marker				
Negative	0.559 (0.389–0.803)	0.002	0.675 (0.468–0.972)	**0.035**
Positive	Reference			
pT‐stage				
pT1	0.196 (0.062–0.622)	0.006		
pT2	0.328 (0.2–0.54)	0		
pT3	0.484 (0.349–0.672)	0		
pT4	Reference			
pN‐stage				
pN0	0.163 (0.111–0.239)	0	0.227 (0.15–0.344)	**0**
pN1	0.428 (0.299–0.612)	0	0.519 (0.357–0.754)	**0.001**
pN2	Reference			
M‐stage				
M0	0.159 (0.109–0.231)	0	0.252 (0.17–0.375)	**0**
M1	Reference			
Vascular invasion				
Negative	0.435 (0.319–0.593)	0		
Positive	Reference			
Nerve invasion				
Negative	0.626 (0.462–0.848)	0.003		
Positive	Reference			
Obstructive				
Negative	0.662 (0.415–1.056)	0.083		
Positive	Reference			
Tumor size				
< 3.8	0.575 (0.419–0.79)	0.001		
≥ 3.8	Reference			
Location of cancer				
Colon	1.17 (0.863–1.587)	0.311		
Rectum	Reference			
Differentiation				
Well	0.274 (0.183–0.412)	0	0.377 (0.248–0.574)	**0**
Moderately	0.466 (0.319–0.681)	0	0.552 (0.374–0.813)	**0.003**
Poorly	Reference			

*Note:* Bold values indicate significance (*p* ≤ 0.05).

Abbreviations: CI, confidence interval; HR, hazard ratio.

#### 3.2.2 Role of CHE in Survival

Among the 1140 CRC patients included in the study, 165 (14.5%) were classified into the CHE^Low^ group, while 975 (85.5%) were classified into the CHE^High^ group. Survival rates were significantly lower in CHE group^Low^ (73.9%) versus the CHE^High^ group (87.3%) (*p* < 0.001; Figure 2B).

#### 3.2.3 CHE in CRC Patients With TMN

In patients with TMN CRC, CHE levels showed significant associations with NLR, LMR, PLR, and pT‐stage (*p* = 0.008, *p* < 0.001, *p* = 0.001, and *p* < 0.001, respectively; Table 3).

**TABLE 3:** Clinicopathological factors of CHE in CRC with TMN.

Variables	CHE		χ2	*p*
	Low (*n* = 48) (13.1%)	High (*n* = 321) (86.9%)		
Gender			0.119	0.755
Male	27 (12.5%)	189 (87.5%)		
Female	21 (13.7%)	132 (86.3%)		
Age			3.977	0.072
< 70	34 (11.3%)	266 (88.7%)		
≥ 70	14 (20.3%)	55 (79.7%)		
BMI			3.85	0.073
< 18.5	7 (25%)	21 (75%)		
≥ 18.5	41 (12%)	300 (88%)		
MMR			1.503	0.244
pMMR	42 (14%)	257 (86%)		
dMMR	6 (8.6%)	64 (91.4%)		
NLR			8.155	0.008
< 3.3	32 (10.6%)	269 (89.4%)		
≥ 3.3	16 (23.5%)	52 (76.5%)		
LMR			14.213	0
< 3	24 (23.8%)	77 (76.2%)		
≥ 3	24 (9%)	244 (91%)		
PLR			12.325	0.001
< 230.5	30 (10%)	269 (90%)		
≥ 230.5	18 (25.7%)	52 (74.3%)		
pT‐stage			19.714	0
pT1	0 (0%)	25 (100%)		
pT2	3 (3.4%)	84 (96.6%)		
pT3	30 (15.4%)	165 (84.6%)		
pT4	15 (24.2%)	47 (75.8%)		
pN‐stage			0.136	0.946
pN0	31 (13.2%)	203 (86.8%)		
pN1	11 (12%)	81 (88%)		
pN2	6 (14%)	37 (86%)		
M‐stage			0.268	0.642
M0	46 (12.8%)	312 (87.2%)		
M1	2 (18.2%)	9 (81.8%)		
Vascular invasion			0.11	0.837
Negative	39 (12.7%)	267 (87.3%)		
Positive	9 (14.3%)	54 (85.7%)		
Nerve invasion			0.125	0.747
Negative	30 (12.6%)	209 (87.4%)		
Positive	18 (13.8%)	112 (86.2%)		
Obstructive			5.923	0.025
Negative	41 (11.9%)	304 (88.1%)		
Positive	7 (29.2%)	17 (70.8%)		
Tumor size			13.618	0
< 3.8	14 (7%)	185 (93%)		
≥ 3.8	34 (20%)	136 (80%)		
Location of cancer			9.549	0.003
Colon	33 (18.6%)	144 (81.4%)		
Rectum	15 (7.8%)	177 (92.2%)		
Differentiation			5.851	0.054
Well	18 (9.6%)	170 (90.4%)		
Moderately	21 (14.8%)	121 (85.2%)		
Poorly	9 (23.1%)	30 (76.9%)		

In addition, Kaplan–Meier analysis demonstrated significant differences among patients with TMN, with 321 (87%) patients in the CHE^High^ group and 48 (13%) patients in the CHE^Low^ group. The survival rate was (79.2%), which was worse than that in the CHE^High^ group (91.3%, *p* < 0.008; Figure 2C). Overall survival was significantly associated with CHE levels in TMN patients.

#### 3.2.4 Association Between TMN and OS in CRC Patients: Results From Univariate and Multivariate Analyses

Cox analyses were conducted to assess the prognostic significance of TMN. In the univariate analysis, significant prognostic factors included age (HR 0.471, 95% CI: 0.236–0.937, *p* = 0.032), BMI (HR 2.891, 95% CI: 1.269–6.587, *p* = 0.012), MMR (HR 4.563, 95% CI: 1.098–18.969, *p* = 0.037), CHE (HR 2.611, 95% CI: 1.263–5.395, *p* = 0.01) and pN1‐stage (HR 0.391, 95% CI: 0.175–0.873, *p* = 0.022). In multivariate analysis, BMI (HR 3.13, 95% CI: 1.35–7.26, *p* = 0.008), CHE (HR 2.71, 95% CI: 1.29–5.69, *p* = 0.008), and pN‐stage (HR 0.35, 95% CI: 0.15–0.78, *p* = 0.01) as independent prognostic factors (Table 4).

**TABLE 4:** Univariate and multivariate analyses of TMN and survival in CRC.

Variables	Univariate analysis		Multivariate analysis	
	HR (95% CI)	*p*	HR (95% CI)	*p*
Gender				
Male	1.858 (0.921–3.747)	0.083		
Female	Reference			
Age				
< 70	0.471 (0.236–0.937)	0.032		
≥ 70	Reference			
BMI				
< 18.5	2.891 (1.269–6.587)	0.012	3.13 (1.35–7.256)	**0.008**
≥ 18.5	Reference			
MMR				
pMMR	4.563 (1.098–18.969)	0.037		
dMMR	Reference			
NLR				
< 3.3	0.828 (0.379–1.81)	0.636		
≥ 3.3	Reference			
LMR				
< 3	1.437 (0.722–2.864)	0.302		
≥ 3	Reference			
PLR				
< 230.5	0.792 (0.362–1.734)	0.56		
≥ 230.5	Reference			
CHE				
≤ 5.4	2.611 (1.263–5.395)	0.01	2.713 (1.293–5.694)	**0.008**
> 5.4	Reference			
pT‐stage				
pT1	0.329 (0.075–1.446)	0.141		
pT2	0.17 (0.055–0.529)	0.002		
pT3	0.414 (0.205–0.836)	0.014		
pT4	Reference			
pN‐stage				
pN0	0.18 (0.083–0.393)	0	0.149 (0.068–0.33)	**0**
pN1	0.391 (0.175–0.873)	0.022	0.345 (0.153–0.779)	**0.01**
pN2	Reference			
M‐stage				
M0	0.238 (0.08–0.709)	0.01		
M1	Reference			
Vascular invasion				
Negative	0.382 (0.193–0.755)	0.006		
Positive	Reference			
Nerve invasion				
Negative	0.672 (0.353–1.278)	0.226		
Positive	Reference			
Obstructive				
Negative	0.828 (0.254–2.698)	0.754		
Positive	Reference			
Tumor size				
< 3.8	0.822 (0.431–1.568)	0.552		
≥ 3.8	Reference			
Location of cancer				
Colon	0.994 (0.525–1.88)	0.984		
Rectum	Reference			
Differentiation				
Well	0.264 (0.107–0.65)	0.004		
Moderately	0.584 (0.253–1.352)	0.21		
Poorly	Reference			

*Note:* Bold values indicate significance (*p* ≤ 0.05).

Abbreviations: CI, confidence interval; HR, hazard ratio.

#### 3.2.5 CHE in CRC Patients With TMP

Kaplan–Meier analysis demonstrated that among the patients with TMP, there were 654 (84.8%) patients in the CHE^High^ group and 117 (15.2%) patients in the CHE^Low^ group, and the survival rate was (71.8%), which was worse than that of the CHE^High^ group (85.3%) (*p* < 0.001) (Figure 2D).

#### 3.2.6 Association of BCHE Expression With Pan‐Cancer Data From TCGA and GTEx in CRC

**FIGURE 3:**
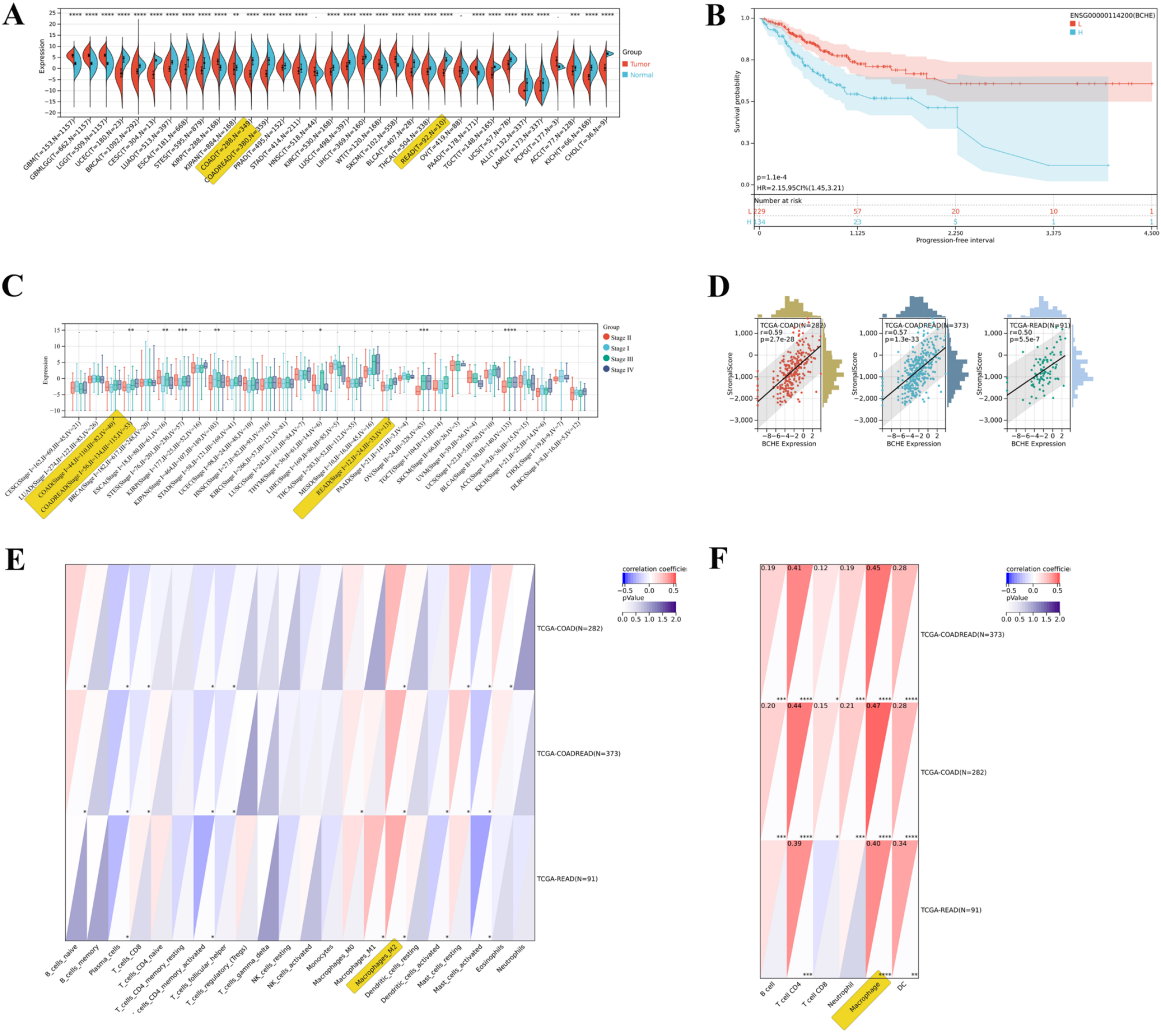
BCHE expression with pan‐cancer data from TCGA and GTEx in CRC. (A) The expression of BCHE in cancer and normal tissues in pan‐cancer. In 34 kinds of cancer, BCHE expression was significantly upregulated in seven tumors and downregulated in 24 tumors. Such as COADREAD (*T* = 380, *N* = 359) statistical significance (*p* < 0.0001). (B) Kaplan–Meier survival analysis of progression‐free survival (PFS) by BCHE expression levels. The patients were stratified into high (H, *n* = 134) and low (L, *n* = 229) BCHE expression groups. The log‐rank test shows significantly worse PFS in the high‐expression group (*p* = 1.1 × 10^−4^; HR = 2.15, 95% CI: 1.45–3.21). Shaded regions around the curves represent the confidence intervals (95% CI). (C) BCHE expression in pan‐cancer and pathological stages observed significant differences in seven tumor types, such as COADREAD (Stage I = 56, II = 134, III = 115, IV = 53) (*p* = 2.2e−3). (D) BCHE gene expression was significantly correlated with immune infiltration, and it significantly positively correlated with the stromal score, *p* = 2.7e−28. (E) QUANTISEQ analysis, the immune cell infiltration (B_cells, Macrophages_M1, Macrophages_M2, Monocytes, Neutrophils, NK_cells, T_cells_CD4, T_cells_CD8, Tregs, dendritic cells (DC), and other cells) was re‐evaluated according to BCHE gene expression. BCHE expression significantly correlates with immune infiltration and is mainly associated with M2 macrophages (*p* = 2.0e−14). (F) TIMER analysis, immune cell infiltration (B cell, T cell CD4, T cell CD8, neutrophil, macrophage, and DC), macrophages show positive correlations (*p* = 2.7e−24) with T cells, B cells, and other immune cells, in BCHE gene suggesting that macrophages might influence or coordinate immune responses in these cancers. Asterisks denote significance (*p < 0.05, **p < 0.01, ***p < 0.001, ****p < 0.0001).

We analyzed pan‐cancer data from TCGA and GTEx to evaluate BCHE expression. In 34 kinds of cancer, BCHE expression was a significant upregulation in seven tumors and downregulation in 24 tumors, such as COADREAD (*T* = 380, *N* = 359) statistical significance (*p* < 0.0001). The details are illustrated in (Figure 3A), and TCGA tumor abbreviations are listed in (Table S1). There is a significant difference in expression levels between tumor and normal samples.

As shown in (Figure 3B), Kaplan–Meier survival analysis of progression‐free survival (PFS) by BCHE expression levels. The patients were stratified into high (H, *n* = 134) and low (L, *n* = 229) BCHE expression groups. The log‐rank test shows significantly worse PFS in the high‐expression group (*p* = 1.1 × 10^−4^; HR = 2.15, 95% CI: 1.45–3.21). This suggests that BCHE expression levels may serve as a prognostic biomarker for disease progression (Table S2).

In 30 cancer types, as shown in the (Figure 3C). The pathological stages observed significant differences in seven tumor types, such as COADREAD (Stage I = 56, II = 134, III = 115, IV = 53) (*p* = 2.2e−3) (Table S3). Suggesting a potential association between BCHE levels and cancer progression.

BCHE gene expression was significantly correlated with immune infiltration, and it significantly positively correlated in the stromal, immune, and ESTIMATE scores, *p* = 2.7e−28, *p* = 7.3e−10, and *p* = 4.4e−20, respectively (Figure 3D, Table S4, Figure S4).

In QUANTISEQ, as depicted in (Figure 3E), the immune cell infiltration (B_cells, Macrophages_M1, Macrophages_M2, Monocytes, Neutrophils, NK_cells, T_cells_CD4, T_cells_CD8, Tregs, Dendritic_cells (DC), and other cells) was re‐evaluated according to BCHE gene expression. BCHE expression significantly correlates with immune infiltration and is mainly associated with M2 macrophages (*p* = 2.0e−14) (Table S5).

In TIMER, immune cell infiltration (B cell, T cell CD4, T cell CD8, Neutrophil, Macrophage, and DC), macrophages show positive correlations (*p* = 2.7e−24) with T cells, B cells, and other immune cells in the BCHE gene, suggesting that macrophages might influence or coordinate immune responses in these cancers, as depicted in (Figure 3F, Table S6).

## 4 Discussion

This study demonstrated that serum CHE levels are valuable prognostic indicators for nutrition‐related serum markers [1]. Synthesized in the liver, serum CHE is the most abundant protein found in blood plasma [20]. Its levels are commonly used to evaluate patients’ nutritional status. Low serum CHE levels have been linked to reduced survival rates in patients with advanced cancer [1]. According to existing literature, CHE activity is diminished in prostate cancer patients, and a persistent decline in these levels may promote the proliferation of prostate cancer cells [21]. This could provide an indirect explanation for the poor prognosis observed in patients with low CHE levels.

Tumor markers are biochemical substances released by tumor cells either as a result of the malignant process or as a cause. These markers can be natural products that are produced in higher quantities by cancer cells, or they can be products of genes that were previously inactive in normal cells but have now become active. When the tumor produces a tumor marker and reaches a significant level, it indicates the presence of cancer. These markers can be found within cells or released into the bloodstream and detected in the serum [2].

CHE deficiency is commonly observed in CRC. An important question that arises is determining who or what is responsible for the negative outcome. To find a solution to this problem, it is crucial to establish a parameter that distinguishes the individuals responsible for the adverse result from those who are not. A method that has gained popularity involves using tumor markers as a threshold or cutoff point. These tumor biomarkers, which are specific molecules or substances found in the serum, can help differentiate patients who are more likely to experience the harmful effects of CHE deficiency from individuals who are less likely to be impacted. By utilizing this method, it becomes possible to identify the individuals responsible for the undesired outcome associated with low CHE in CRC.

Research indicates that low levels of CHE are linked to increased mortality rates in critically ill patients [22]. Specifically, diminished serum CHE levels heighten the risk of death in various medical conditions, including acute myocardial infarction [23], acute heart failure [24], acute respiratory distress syndrome [25], stable coronary artery disease [26], gastric cancer [27], and ischemic stroke [28]. Some studies have shown that low preoperative serum CHE levels serve as an independent risk factor for postoperative complications in elderly patients undergoing emergency major gastroenterological surgeries [29].

BCHE is emerging as a promising biomarker for cancer diagnosis [30]. Reduced BCHE activity in blood plasma is associated with shorter survival times in pancreatic cancer patients [31], and it has been observed to have low expression levels in colorectal carcinoma [32] while exhibiting high expression in oral cancer [33] and ovarian cancer [34]. In children with hand, foot, and mouth disease caused by EV71, CHE activity is higher compared to healthy controls [35]. Furthermore, in prostate cancer, BCHE expression decreases in the early stages but increases during advanced stages [21]. This article highlights the varying expressions of BCHE in CRC using multiple publicly available databases. To elucidate BCHE's role in poor CRC outcomes through integrated visualization with matched clinical data.

Overall, BCHE is recognized as a marker of liver function and is also used to assess nutritional status routinely [10]. In the realm of CRC, it has been observed that there exist divergences in the manifestation of BCHE between cancerous tissues and normal tissues [12]. Moreover, it has been found that the expression of BCHE is notably linked to the pathological stage as well as the progression‐free survival of the disease [36, 37].

Analysis of immune infiltration showed a positive relationship between BCHE expression and the presence of immune cells, particularly M2 macrophages. BCHE appears to play a predictive role in the survival of cancer patients and is associated with disease progression. In this context, serum BCHE levels serve as an effective functional and prognostic indicator, aiding the monitoring of clinical and therapeutic interventions based on patient survival expectations [30]. The enhanced activity of CHE, such as BCHE, is thought to contribute to systemic inflammation by disrupting the cholinergic anti‐inflammatory pathway through the hydrolytic breakdown of acetylcholine [38]. Moreover, lower CHE levels have been linked to poorer outcomes, including increased mortality or worsening clinical conditions in patients with CRC.

## 5 Limitations of the Study

Due to the retrospective study, the main limitation was the lack of specimen availability, which may affect the accuracy of identifying key biomarkers, immune responses, and potential confounders. Additionally, unequal sample sizes between the low and high CHE level groups could reduce statistical power and introduce bias. Future studies with larger, balanced cohorts are needed.

## 6 Conclusion

In CRC with TMN, CHE^Low^ is an independent detrimental prognostic factor for OS. BCHE may serve as a biomarker for CRC to help predict its prognosis and may have an important impact on immunotherapy.

## Author Contributions

**Tawfik Ali Hamood Alburiahi:** data curation (lead), formal analysis (lead), methodology (lead), writing – original draft (lead). **Lei Liang:** data curation (equal), formal analysis (equal). **Weiqing Liu:** data curation (supporting), formal analysis (supporting). **Zhiyong Kou:** data curation (supporting). **Yunfei Zhang:** data curation (supporting). **Ning Xu:** investigation (supporting), methodology (supporting). **Jun Yang:** conceptualization (lead), investigation (lead), methodology (lead), supervision (lead), writing – review and editing (lead).

## Ethics Statement

The study design was approved by the Ethics and Human Subject Committee of the First Affiliated Hospital of Kunming Medical University, and a waiver of patient informed consent was granted (Ethics Approval No. (2023) Ethics Approval L No. 150).

## Conflicts of Interest

The authors declare no conflicts of interest.

## Supporting information


**Data S1.** Supporting Figures.

**Table S1.** The full names of TCGA tumor abbreviations.

**Table S2.** Progression‐free survival of BCHE in colorectal cancer, including 134 cases with high BCHE expression and 229 cases with low expression.

**Table S3.** The expression difference of genes in each tumor in samples of different clinical stages.

**Table S4.** A positively correlated of BCHE gene in immune infiltration score.

**Table S5.** QUANTISEQ, the immune cell infiltration in BCHE gene.

**Table S6.** TIMER, Immune cell infiltration in BCHE gene.

**Table S7.** Association analysis of clinicopathological factors and OS after CHE low and CHE high 1:1 match.

## Data Availability

The data supporting the findings of this study are available from the corresponding author upon reasonable request.
