# Supplementary material for: Low Cholinesterase Is a Potential Poor Prognostic Factor in Colorectal Cancer Presenting With Tumor Markers Negative
Source: Cancer Rep (Hoboken). 2025 Aug 1;8(8):e70266. doi: 10.1002/cnr2.70266 (PMC12317114; doi:10.1002/cnr2.70266)
Supplement: Supplementary file 1 — Data S1. Supporting Figures. [file CNR2-8-e70266-s004.pdf]

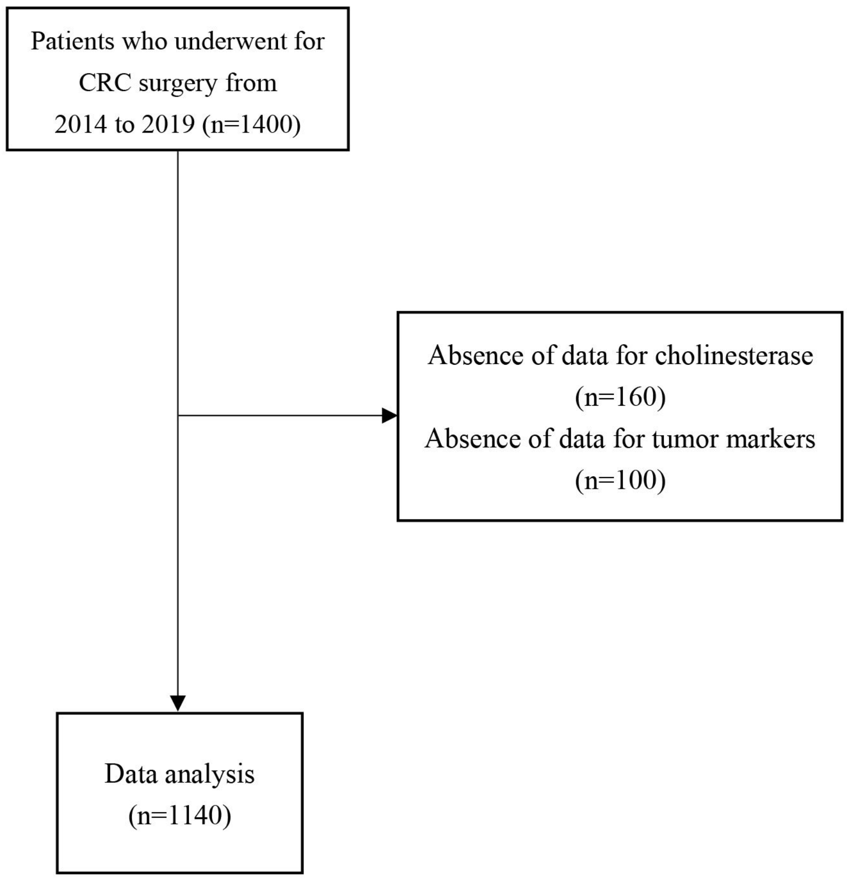

Supplementary Figure S1. Flowchart illustrating the patient cohort selection process based on inclusion and exclusion criteria.

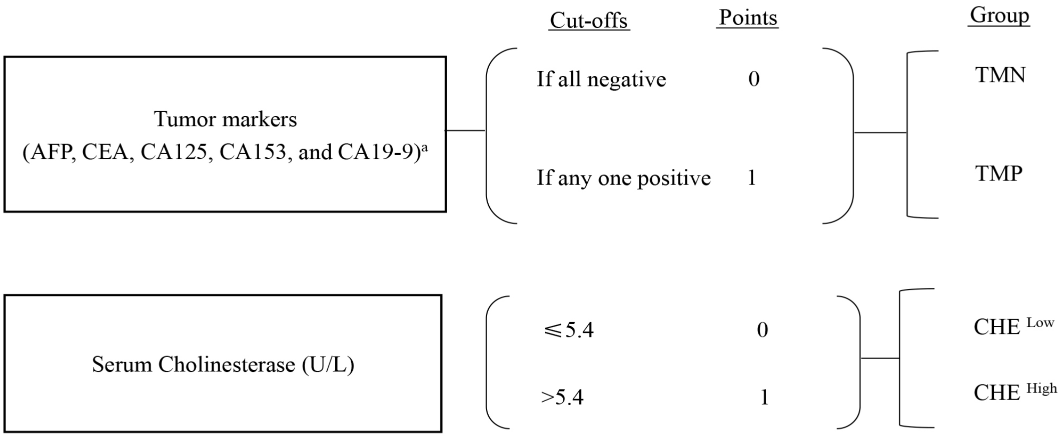

Supplementary Figure S2. Flowchart for identifying the study. a, (AFP) Alpha-Fetoprotein, (CEA) carcinoembryonic antigen, (CA125) carbohydrate antigen 125, (CA153) carbohydrate antigen 153 and (CA 19-9) carbohydrate antigen 19-9.

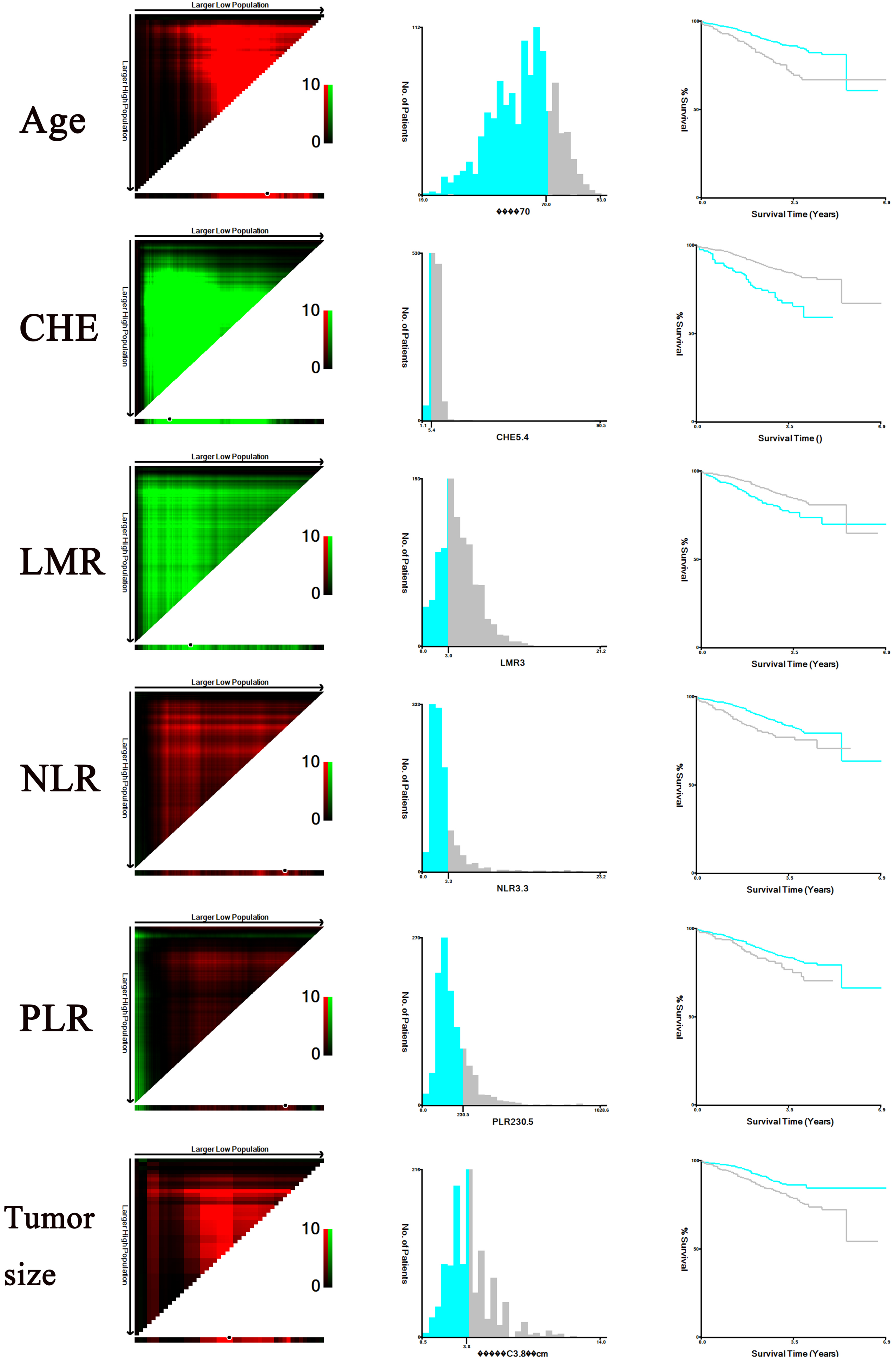

Supplementary Figure S3. The cut-off of continuous variables.
The cut-off of age, cholinesterase (CHE), lymphocyte-to-monocyte ratio (LMR), neutrophil-to-lymphocyte ratio (NLR), platelet-to-lymphocyte ratio (PLR), and tumor size are 70, 5.4 U/L, 3, 3.3, 230.5, and 3.8cm.

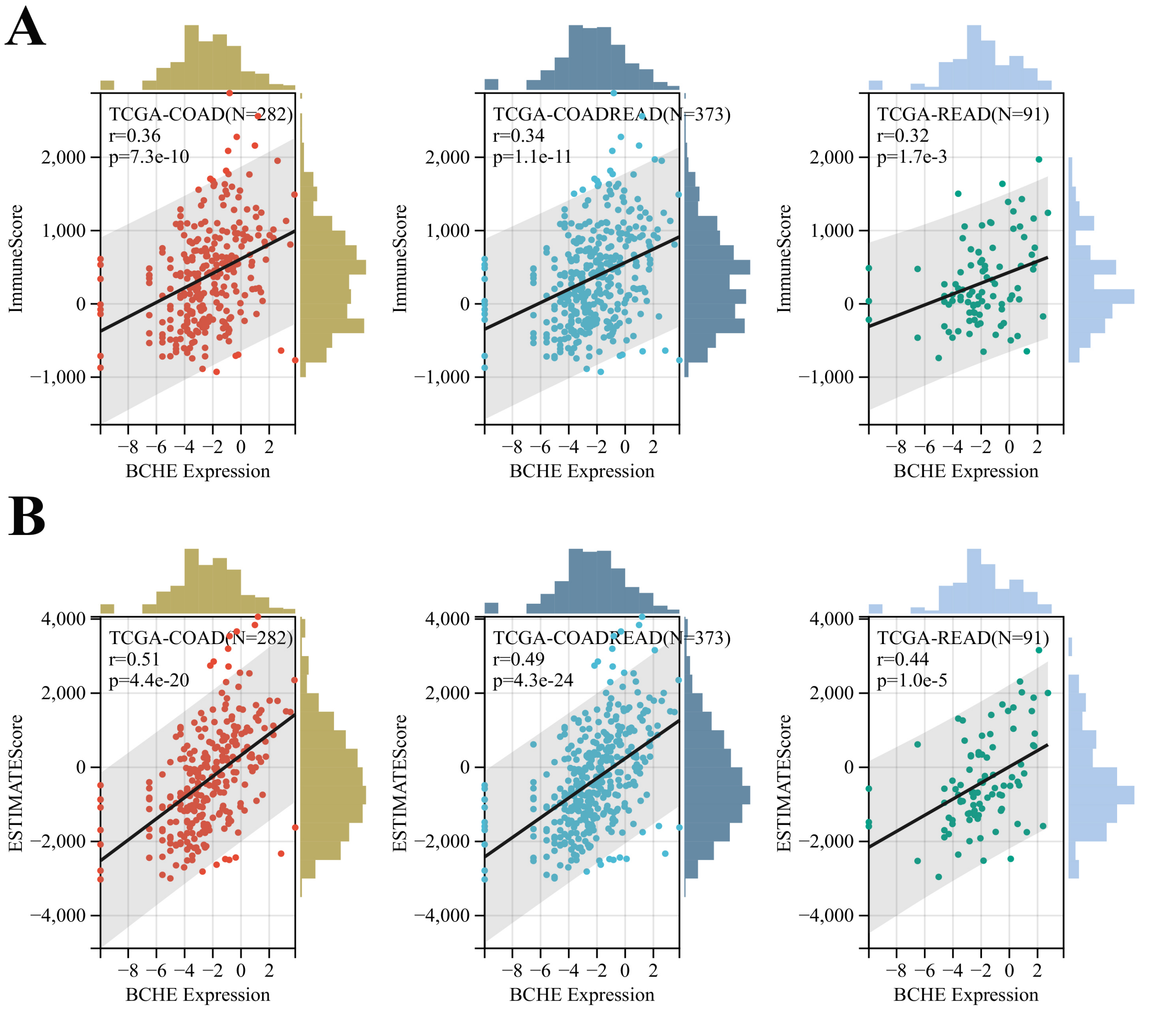

Supplementary Figure S4.
A BCHE gene expression was significantly correlated with immune infiltration, and it significantly positively correlated with the immune score, P=7.3e-10.
B BCHE gene expression was significantly correlated with immune infiltration, and it significantly positively correlated with the ESTIMATE score, P=4.4e-20.

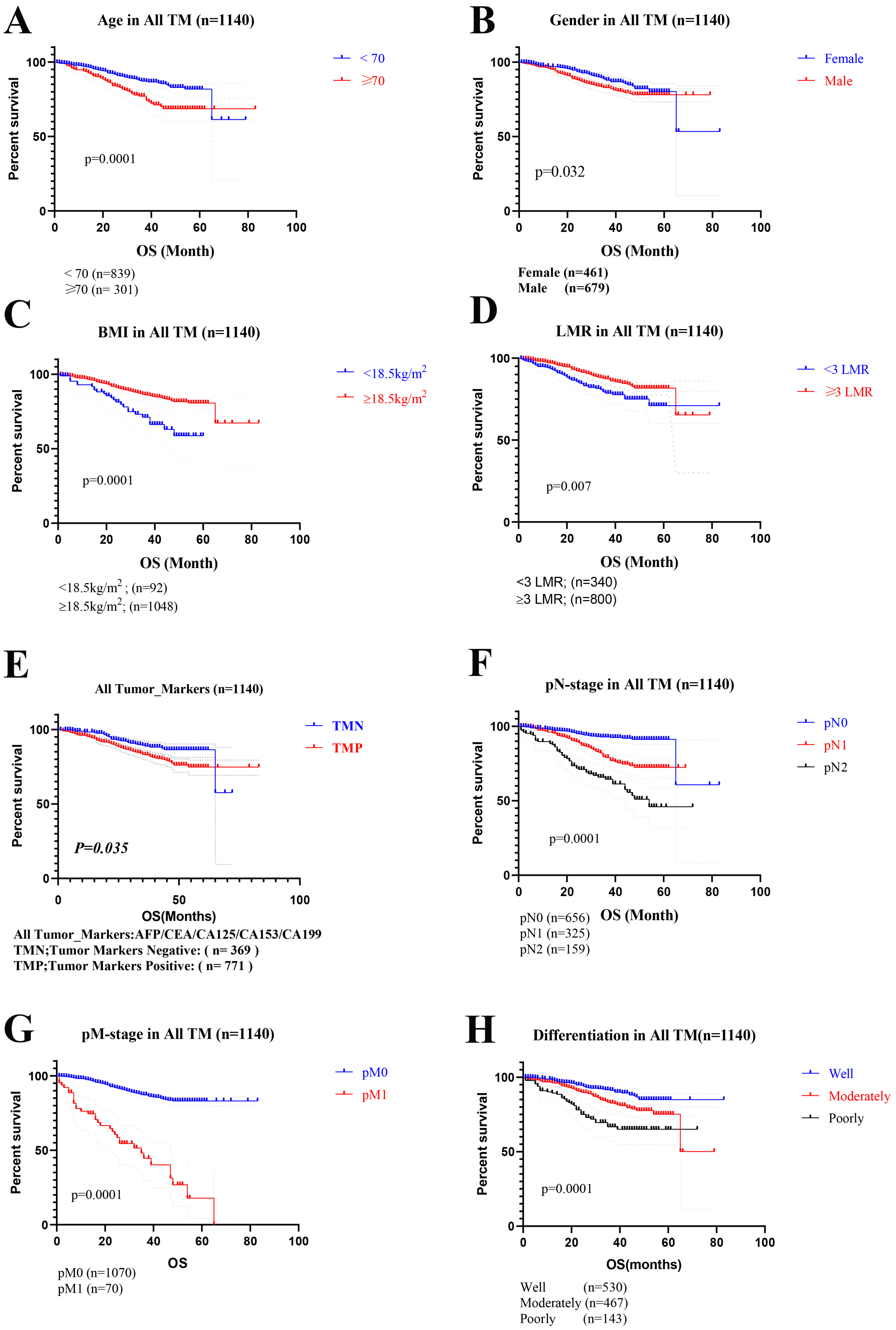

Supplementary Figure S5. Survival prognosis of all tumor markers in colorectal cancer. (A-H) Kaplan-Meier curves of overall survival (OS) based on age, gender, BMI, LMR, pN-stage, pM-stage, and differentiation. All tumor markers show significant associations with survival outcomes.

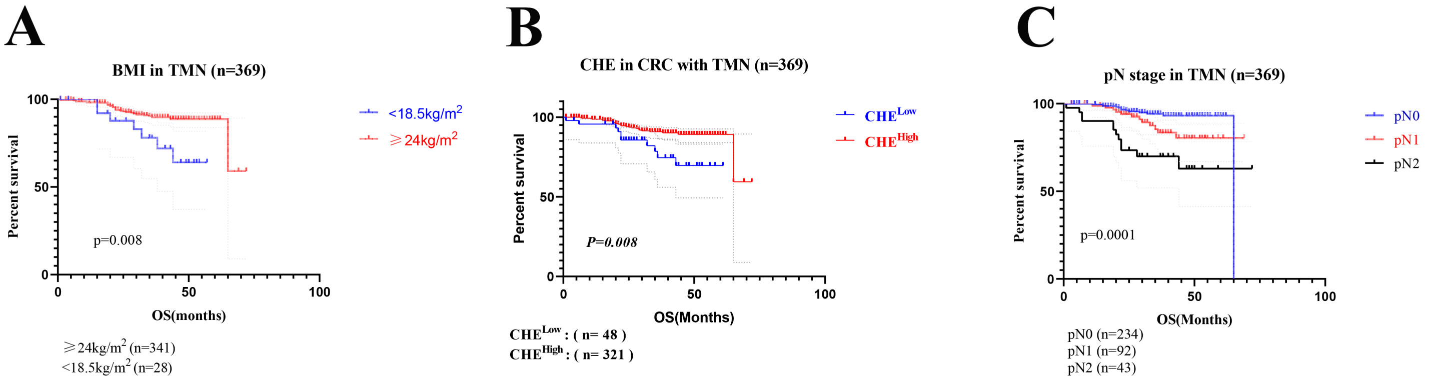

Supplementary Figure S6. Survival prognosis of tumor marker-negative (TMN) cases in colorectal cancer. (A–C) Kaplan-Meier curves of overall survival (OS) based on BMI, CHE, and pN-stage. TMN status shows significant associations with survival outcomes.
